# Quantitative Trait Loci Mapping

**Published:** 1995

**Authors:** Judith E. Grisel, John C. Crabbe

**Affiliations:** Judith E. Grisel, Ph.D., is a postdoctoral fellow and John C. Crabbe, Ph.D., is both a research career scientist at the Department of Veterans Affairs Medical Center and a professor at the School of Medicine, Oregon Health Sciences University, Portland, Oregon

**Keywords:** AOD use behavior, animal strains, hereditary factors, genetic mapping, genetic markers

## Abstract

Researchers interested in the physical locations of genes that influence a person’s alcohol-related behaviors can use a method known as quantitative trait loci (QTL) mapping to identify the approximate locations of genes in the genome. QTL mapping can use recombinant inbred mouse strains, which are sets of inbred strains derived from cross-breeding the offspring of two genetically distinct parent strains. The inbred strains exhibit different patterns of the parent strains’ genes. QTL mapping involves comparing alcohol-related behaviors in these strains and identifying patterns of known genetic markers shared by strains with the same behaviors. The markers allow the identification of probable locations of genes that influence alcohol-related behaviors. These locations can then be verified using other tests, and specific genes can be sought there.

Alcohol’s complex impact on behavior is demonstrated by studies that attempt to relate problems associated with alcohol abuse, such as dependence on alcohol or tolerance to its effects, to the biological and molecular mechanisms underlying those effects. Alcoholism^1^ has been described as a polygenic disorder, that is, one that is influenced by many genes located in different areas, or loci, of a person’s or animal’s DNA (McClearn et al. 1991; Goldman 1993). The genetically influenced characteristics, or traits, thought to underlie responses to alcohol (e.g., sensitivity to its effects) are called quantitative traits, and many genes influence the overall characteristic, each to a certain extent. Thus, within a population, a quantitative trait differs in the degree to which individuals possess it (e.g., height) rather than in the kind of trait they possess (e.g., eye color). Accordingly, a section of DNA thought to contribute to a quantitative trait is called a quantitative trait locus (QTL). Susceptibility to cancer is another example of quantitative traits that are determined by the combined contributions of several QTLs. Quantitative traits are said to be continuously distributed in a population, because individuals exhibit them to different degrees. Because of this distribution, quantitative traits are much more difficult to study than qualitative traits, such as eye color or blood type, which are determined by a single locus and which therefore are distributed discretely.

One way to study the contributions of individual QTLs to a quantitative trait such as sensitivity to alcohol is for researchers first to locate in the genome (i.e., an organism’s entire genetic material) the genetic information that encodes these traits. A technique for finding this information is called QTL mapping. After a QTL has been identified, the gene can be isolated and its functions studied in more detail. Thus, QTL analysis provides a means of locating and measuring the effects of a single QTL on a trait, or phenotype.^2^ This article provides a brief overview of the methods involved in QTL mapping. It also discusses the advantages and limitations of QTL analysis and includes several examples demonstrating the application of the technique.

## Mapping the Genome

**Figure 1:**
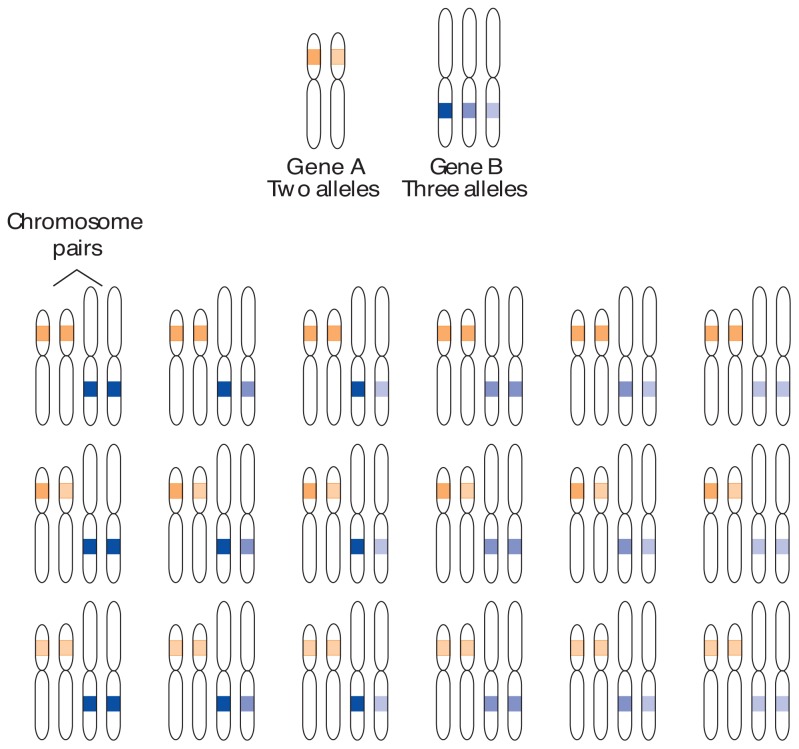
Quantitative traits are determined by more than one gene, each of which can exist in several variants, or alleles. In this example, a particular trait is determined by two genes. Gene A has two alleles, and gene B has three alleles. A person inherits 23 chromosome pairs; one-half of the pair comes from the father, and the other from the mother. Each pair contains alleles of the same genes; one copy of the gene is on each chromosome. The gene pairs may be identical or different. For the hypothetical trait illustrated here, 18 different genotypes exist. If additional genes with more alleles contribute to a trait, the number of possible genotypes increases exponentially, and the trait is said to be continuously distributed.

A QTL is a small section of DNA on a chromosome thought to influence a specific trait. Scientists search different areas of the genome for locations (i.e., loci) they can associate with the trait. The gene included in each QTL exists in more than one form, or allele, and can differ between individuals in a population. One person can carry two different alleles of a gene, one inherited from the mother and one from the father. The effect of one QTL is often fairly small. Thus, the collective impact of many genes located at several QTLs provides the genetic influence on different behavioral and physical phenotypes, such as those related to alcohol abuse (figure 1).

For technical reasons, QTL mapping for alcohol-related traits is more commonly performed in animal models than in humans; however, because of the common evolutionary history of all mammals, large regions of our genomes have a common identity. This method is therefore a potentially rich source of information about the genes associated with alcohol abuse in humans (Copeland et al. 1993).

### Genetic Markers

To identify the location of QTLs, researchers determine the degree to which a phenotype, such as a person’s initial sensitivity to alcohol’s effects, is associated with a known genetic marker. These markers are DNA segments known to occupy particular places on the chromosomes.^3^ Each marker is polymorphic—that is, it exists in several different variants that can distinguish individuals or strains of laboratory animals from one another (for a further discussion of genetic markers, see the article by Anthenelli and Tabakoff, pp. 176–181). The association of a genetic marker with a certain trait allows researchers to estimate both the location of a QTL and the magnitude of its contribution to the trait (Tanksley 1993). For example, if all persons with one specific marker are particularly sensitive to alcohol, then this marker constitutes, or is located close to, a QTL contributing to the trait “sensitivity.”

A marker can be a gene, but it also may be derived from a region of the genome that does not produce a functional product, that is, does not underlie the production of proteins. In the latter case, researchers assume that the marker is located near an un-mapped gene. Information on these marker locations is helpful in finding new genes. Advances in molecular biology in the past 15 years have led to the identification of thousands of genetic markers; increasingly dense maps are being constructed for human, mouse, and rat genomes using QTL and related mapping techniques.

## BXD Recombinant Inbred Strains

**Figure 2:**
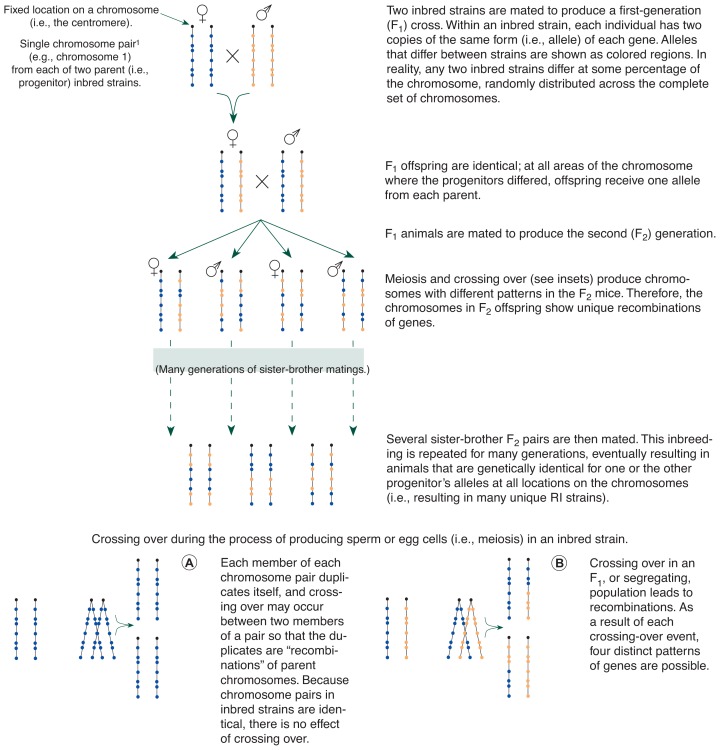
The process of deriving recombinant inbred (RI) strains. An example for one chromosome pair is shown. Two parent inbred strains are depicted. Although DNA with multiple forms is distributed throughout the entire set of an animal’s genetic material, for the purposes of illustration, DNA specific to one is shown in blue, and DNA specific to the other is depicted in orange (shared regions are left uncolored). ^1^Chromosomes are lengths of DNA that contain genes and compose most organisms’ genetic material. Chromosomes in cells are paired. One chromosome of the pair is inherited from the organism’s father, and the other is inherited from the mother.

Most QTL analyses are performed in laboratory animals, specifically in mice. For their experiments, researchers prefer to compare different strains of mice in which all animals of a strain have identical genetic material. One type of animal fulfilling this requirement is the so-called recombinant inbred (RI) mouse strain. RI strains are derived from a population of second generation, or F_2_, offspring of two genetically distinct parental, or progenitor, inbred strains (Bailey 1971). In the RI method, the members of a single strain are inbred to be genetically identical (i.e., they all have the same alleles at all loci). Figure 2 shows, for a single chromosome pair, the process by which RI strains are developed.

Researchers have extensively used one such group of animals, the BXD RI strains, to investigate the genes that influence alcohol-related effects. These RI strains are derived from the F_2_ offspring of two progenitor strains called C57BL/6J (C57) and DBA/2J (DBA). Through recombination of the genetic material, each F_2_ mouse inherits a distinctive combination of genes from the two progenitor strains. Sister-brother pairs of F_2_ mice are mated to begin to “fix” this unique pattern of recombinations (see figure 2). Their progeny are inbred for many more generations, ultimately resulting in an RI strain of mice that are identical (homozygous) at each locus. For any gene, each mouse possesses two copies of the allele from either the C57 or the DBA progenitor.

Several different strains can be generated by inbreeding different sister-brother pairs from the original group of F_2_ animals. Such a family, or set, of RI strains allows researchers to compare strains that each have distinct patterns of the same marker set in their genomes.

Currently, 26 BXD RI strains exist, each with a unique genotype. These animals are especially useful for studying the effects of alcohol because their progenitors, the C57 and DBA mice, differ widely with respect to many alcohol-related traits. Other RI sets exist, but only the long-sleep x short-sleep (LS x SS) set, discussed below, also has been used to study alcohol-related phenotypes (Markel et al. in press).

### Locating QTLs in RI Strains

QTL mapping correlates the differences in alleles of particular genetic markers with differences in phenotypes in a population. The methodology relies on statistical correlations. In the C57 and DBA mouse strains and in most of the BXD RI strains derived from them, for example, researchers have identified numerous markers. Alcohol-related phenotypes also have been ascertained and recorded for both of the progenitor strains and the battery of BXD RI’s. All this information has been collected in a database. By comparing the phenotypes for each strain with the pattern of genetic markers for that strain, researchers can determine if relationships exist between particular phenotypes and markers.

**Figure 3:**
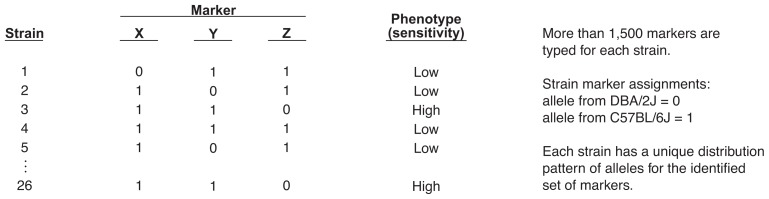
A chart relating markers X, Y, and Z and strain traits (i.e., phenotypes). Strains 3 and 26 have a phenotype of high sensitivity to alcohol’s effects. These mice have the form of a gene (i.e., an allele) from the C57BL/6J parent at markers X and Y and an allele from the DBA/2J parent at marker Z. In this manner, markers correlated with the phenotype can be identified.

For example, assume that every strain possessing a DBA allele of markers X and Y and a C57 allele of marker Z on the genome shows high sensitivity to alcohol’s depressant effects (figure 3). Strains with C57 alleles at X and Y and a DBA allele at Z, in contrast, show low sensitivity to these effects. Thus, the pattern of alleles at these loci predicts alcohol sensitivity. At other loci, whether an animal possesses a DBA or C57 allele may not affect sensitivity; therefore, no significant correlation would exist for these alleles. Using statistical methods, correlation coefficients^4^ are obtained for each phenotype and for each marker that has a different allele for C57 and DBA mice. High correlation coefficients, when statistically significant, indicate that the phenotype may be influenced by the genetic information at this particular locus.

One key to successful QTL mapping is the identification of a large array of markers that are well distributed over all 20 mouse chromosomes. As mentioned previously, the strength of the BXD RI strains comes primarily from the large number of markers that have been identified for them, more than 1,500 to date. QTL analysis also is being conducted in less densely mapped RI sets, but the statistical procedures in these cases are somewhat more complex (Johnson et al. 1992; Markel et al. in press).

### Applying QTL Analysis

The association of a marker with a certain phenotype does not necessarily mean that the marker *is* the gene contributing to the phenotype. The marker may just be located close enough to the QTL that it is not separated by genetic recombination processes (for more information on genetic recombination and linkage, see sidebar, p. 224). Ultimately, researchers hope to find markers ever closer to the locus that actually produces an effect on a phenotype until a gene that influences the trait eventually is identified. Although the process appears lengthy and tedious, QTL studies begin with no information about the genes in question and rapidly narrow the location to a relatively small piece of DNA. The procedure is like looking for a single family in the entire United States, even narrowing its location to a particular county is extremely helpful to the search effort.

**The Role of Linkage in Inheritance**

One basic concept underlying quantitative trait loci analysis is that of linkage, or the tendency of genes near each other on a chromosome to be inherited together. When cells undergo meiosis (i.e., the specialized process of cell division that generates sperm and egg cells), genetic material on one chromosomal strand changes places with the corresponding material on its pair strand, a process called crossing over (see figure 2 in text).^1^ Genes that are on different chromosomes are inherited independently of each other, whereas genes from the same chromosome generally show increasing dependence the closer they are to one another along the length of the chromosome. Consequently, genes near each other are more likely to remain together after crossing over than those farther from each other, because distance increases the chances that the chromosomal break will occur between the two genes in question. For mapping studies, the unit of chromosome length, the centimorgan (cM), is defined as the length of chromosome within whose span a 1-percent likelihood exists of crossing over.

— *Judith E. Grisel and John C. Crabbe*

1Crossing over occurs only between chromosome pairs, which contain the DNA encoding the same set of genes. Thus, when pieces of a chromosome change places, each chromosome may receive new forms (i.e., alleles) of certain genes, but neither chromosome loses any genetic information.

Once QTL analyses have identified a chromosome region containing a gene that may affect a certain phenotype (i.e., a candidate gene), other tests can be performed to determine the magnitude of the gene’s influence. For instance, researchers can use pharmacological or molecular methods to alter the protein that the gene produces and then measure the effects of this manipulation on the phenotype (for further discussion of such methods, see the article by Hiller-Sturmhöfel and colleagues, pp. 206–213). The results can then be used to substantiate the gene’s effect.

A study by Crabbe and colleagues (1994*b*) of alcohol-induced hypothermia (i.e., the drop in body temperature that occurs after alcohol administration) provides one example of this kind of QTL analyses. The study found that hypothermic sensitivity to alcohol was associated with the marker *D1Byu7*. This marker is located near a gene called *Ltw-4* on mouse chromosome 1. *Ltw-4* codes for a prevalent protein expressed in brain, liver, and kidney tissues. The *Ltw-4* locus also is associated with the amount of alcohol an animal will drink, amphetamine-induced hyperthermia, and withdrawal from some central nervous system depressants, indicating that a single gene might influence various drug-related phenotypes (Crabbe et al. 1994*b*). The mechanism by which this protein may influence hypothermic sensitivity and other traits currently is not understood, but now that *Ltw-4* has been identified as a candidate gene, these questions can be addressed in more detailed analyses.

### Benefits and Limitations of the RI–QTL Approach

#### Benefits

The RI–QTL approach has numerous advantages. It can provisionally identify candidate genes for a particular phenotype without any prior knowledge of the biological mechanisms that influence such phenotypes. Simply determining the correlation coefficients between known markers and a quantitative trait in several RI strains readily yields information about the possible location of genes influencing that trait. Furthermore, all information obtained in QTL studies, including data on individual animal strains, is both cumulative and comparable, providing a rich resource and incentive for collaborative efforts. This benefit exists because researchers can obtain the computer-stored information, which makes the determination of genetic correlations for different traits possible.

#### Limitations

One unfavorable consequence of analyses in which a large number of correlations are evaluated is that some correlations occur by chance rather than because of a real relationship between a marker and a phenotype. This problem makes further analysis of each QTL necessary (discussed below). Although the statistical tests used to determine correlations can be made more stringent, this strategy may miss some genuine relationships. A more practical approach, therefore, is to use a second population that also has been screened for the proposed QTLs and phenotype to confirm QTLs identified in the first set of animals. Only those associations seen in both analyses are considered to represent QTLs affecting the trait.

One instance of this confirmation approach involves the example of the *Ltw-4* gene mentioned previously. Before *Ltw-4* was implicated as a candidate gene using QTL approaches, Goldman and colleagues (1987) noted an association of the *Ltw-4* locus with alcohol consumption in a set of standard inbred strains. The independent confirmation through QTL analysis of a relationship between this locus and responsive-ness to alcohol in RI strains provided further evidence that *Ltw-4* may be important for influencing alcohol-related phenotypes. A recent study by Rodriguez and colleagues (1995), however, found no association between *Ltw-4* and another measure of alcohol consumption in the BXD RI’s. Although differences in methodology may account for these discrepancies, this example demonstrates the value of QTL analysis for generating hypotheses as well as the need for subsequent testing of those hypotheses in different animal populations and experimental settings. Several other approaches to confirming QTLs have been discussed in recent reviews (Tanksley 1993; Lander and Schork 1994).

Because most RI sets consist of only a limited number of strains, many QTL studies are limited to detecting QTLs with relatively large effects (Belknap 1992). Undoubtedly, important QTLs with smaller effects are missed using this method. In the future, after researchers have identified more of the loci having major effects, the loci with smaller effects will be easier to determine.

Furthermore, QTL analysis assumes that no interactions occur in which some genes alter the expression or function of others; rather, the premise is that each locus affects a phenotype independently of other loci. This limitation can be addressed by producing so-called congenic mice, in which the QTL of interest is bred into a known inbred strain; in this way only a small chromosomal segment containing the potential QTL differentiates the congenic strain from the inbred strain (Bailey 1981). For example, assume that results of QTL analysis suggest that 50 percent of the genetic influence on the phenotype arises from the identified locus, whereas tests in congenic mice demonstrate an influence of only 20 percent. Such a disagreement indicates that other genes, in conjunction with the locus in question, are likely to have influenced the phenotype in the QTL experiment.

## Examples of Applying QTL Analysis

Following are a few examples that illustrate the procedures involved in the QTL approach and demonstrate the potential of these analyses.

### Hypothermia: Several QTLs Determine One Trait

When Crabbe and colleagues (1994*b*) performed QTL analysis to identify loci contributing to alcohol-induced hypothermia, they found a correlation with markers other than *D1Byu7* on chromosome 1 (mentioned above). They also discovered that the animals’ hypothermic sensitivity correlated with two closely associated markers on chromosome 9. This example underscores the strength of the QTL approach for simultaneously identifying genetic loci in completely different regions of the genome that contribute to the same trait.

The two hypothermia-associated markers on chromosome 9 are located in the same region as the gene for a neurotransmitter receptor, the serotonin 5HT_1B_ receptor (Crabbe et al. 1994*b*) (for a discussion of different neurotransmitters, see box). To test the possibility that serotonin activity at 5HT_1B_ receptors plays a role in the hypothermic response, knockout mice (i.e., mice whose genes have been experimentally altered) (for further information on knockout mice, see the article by Hiller-Sturmhöfel and colleagues, pp. 206–213) lacking 5HT_1B_ receptors were evaluated for their sensitivity to alcohol. These mice were found to be less sensitive to alcohol-induced hypothermia than normal mice were, thus providing further support for the hypothesis, generated through QTL analysis, that activity at this receptor might play a role in the hypothermic response to alcohol.

**Alcohol-Related Responses in the Central Nervous System**

Drinking alcohol produces several immediate effects that vary among people, including changes in behavior, such as losing one’s inhibitions or becoming suddenly aggressive. But how do these behavioral changes occur, and why do they differ from person to person? Scientists believe that many individual responses to alcohol originate with genes active in the central nervous system, which controls behavior. Some of these genes are thought to produce substances essential to the brain’s ability to relay messages between nerve cells.

All brain activities depend on cell-to-cell communication. Nerve cells grow long projections during development to connect with each other in a vast and complex network that makes up brain tissue. Signals, the language of the brain, travel along these projections, moving from one cell to the next in the network. Microscopic gaps exist between the ends of many nerve cells’ outgoing projections and the beginnings of other cells’ incoming projections. These gaps are called synapses, and they must be bridged—using chemicals called neurotransmitters—for a signal to travel between nerve cells. Neurotransmitters are believed to be important in many alcohol-related responses, and different forms (i.e., alleles) of genes that encode neurotransmitter molecules could contribute to individual reactions to alcohol.

A nerve cell sending a signal across a synapse usually releases a neurotransmitter into the synaptic space. Across the synapse, the receiving cell has protein molecules called receptors embedded in its message-receiving surface. The receptors are designed to recognize and bind specific neurotransmitters; several types of receptors can exist for a single neurotransmitter. The binding of neurotransmitter molecules to the appropriate receptors sets off a series of chemical reactions inside the receiving nerve cell that ultimately results in information being processed and transmitted in the brain. Some of the neurotransmitters and their receptors that are mentioned in the articles in this issue have the following general functions:

- *Acetylcholine* is a common neurotransmitter that usually excites receiving cells. Its receptor is composed of several building blocks, or subunits. One is the gamma subunit, which is encoded by the *Acrg* gene. This subunit may play a role in several alcohol-related responses.
- *Catecholamines* are a class of neurotransmitters, one of which is *dopamine*. Dopamine is involved in motor, reward, and cognitive functions; emotions; and aggression. Alcohol’s effects on catecholamines may contribute to alcohol tolerance.
- *Gamma-aminobutyric acid (GABA)* and its receptors in the brain inhibit receiving nerve cells. These systems are important for sensory processing and coordination of motor control. GABA may mediate some of alcohol’s effects in the brain.
- *Glutamate* is a protein building block (i.e., an amino acid) that also functions as a neurotransmitter and excites receiving nerve cells. Glutamate activity that occurs at the *N*-methyl-D-aspartate (NMDA) receptor may contribute to alcohol-withdrawal seizures.
- *Serotonin* and its receptors affect mood, sleep, drug consumption, the development of tolerance to alcohol and other drugs, higher cognitive functions, and the sensation of pain. Serotonin activity has been connected with alcohol-related responses. For example, one of serotonin’s receptors, the 5HT_1B_, appears to play a role in the drop in body temperature that occurs after alcohol administration.

— *Kathryn Ingle*

### The *Acrg* Locus: One QTL May Affect Several Traits

Not only can several QTLs influence one trait; one QTL also may affect several traits. One gene that demonstrates this capability is the *Ltw-4* gene discussed earlier. Another is a QTL near the gene *Acrg*, located on chromosome 1, which codes for a portion of the receptor for the neurotransmitter acetylcholine. Rodriguez and colleagues (1995) reported that this locus is associated with several alcohol-related responses. Their QTL analysis indicated, for example, that *Acrg* correlates strongly with alcohol acceptance^5^ across the RI strains.

**Figure 4:**
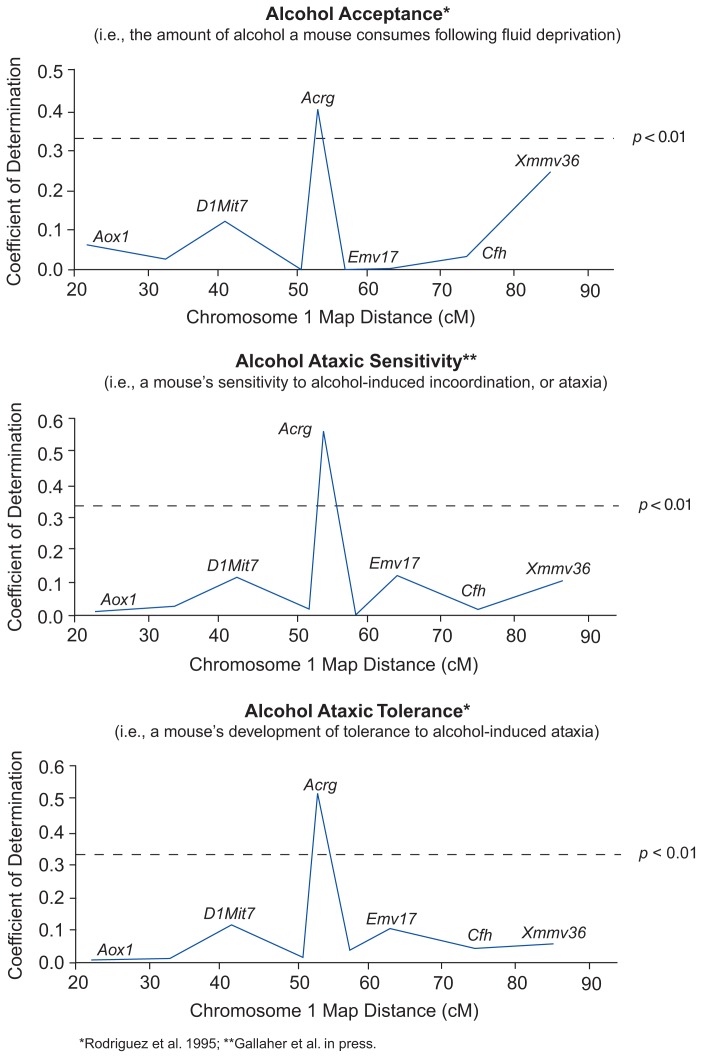
Schematic depiction of mouse chromosome 1. Map distances in centimorgans (cM) from the centromere^1^ (at cM = 0) are shown for several markers, whose names are given in italics (e.g., *Aox1*). The Y-axis (i.e., the coefficient of determination) indicates the proportion of trait (i.e., phenotypic) variance between strains accounted for by each marker. The dotted line indicates the proportion of variance required for statistical significance (*p* < 0.01). To have a significant effect on a phenotype, a location on a chromosome (i.e., a locus) must have a coefficient of determination value higher than the significance line. Three phenotypes are shown; all are significantly associated with the marker *Acrg*. ^1^A centromere is a fixed location on a chromosome that controls the movement of the chromosome during cell division. A centimorgan is the length of the chromosome within whose span there exists a 1-percent likelihood of crossing over (see figure 2, insets).

In separate studies investigating the same QTL, Gallaher and colleagues (in press) demonstrated an association between the *Acrg* locus and a different measure of sensitivity to alcohol, alcohol-induced ataxia (i.e., incoordination of voluntary movements). The *Acrg* locus correlated with the initial ataxic sensitivity to alcohol as well as with the development of the tolerance to ataxia that accrues following repeated alcohol injections (figure 4). The results demonstrated that *Acrg* or a nearby gene may contribute between 40 and 60 percent of the genetic influence on these phenotypes.

Given the concordance in results across two different studies and three phenotypes—alcohol acceptance, initial ataxic sensitivity, and tolerance to ataxia—*Acrg* is likely to be an important QTL. As always, however, this finding should be considered preliminary until a relationship between *Acrg* and these behaviors can be confirmed using other approaches. For example, it is currently unknown whether the part of the acetylcholine receptor encoded by *Acrg* mediates these alcohol effects or whether a nearby gene influences these characteristics. Nevertheless, the combined QTL studies have led to a specific hypothesis, and the *Acrg* locus now can be further evaluated for its influence on alcohol drinking and incoordination.

### Withdrawal: Followup Experiments Confirm the Significance of a QTL

Using QTL methods, researchers have identified several loci that may influence the severity of alcohol withdrawal (Belknap et al. 1993; Crabbe et al. 1994*a*). One of these is a site near the *b* locus on mouse chromosome 4. To confirm the hypothesis that this QTL contributes to the severity of withdrawal, individual F_2_ offspring derived from C57 and DBA parents were tested for acute alcohol withdrawal and the presence of genetic markers in this region. This followup study determined a significant association between alcohol withdrawal and the *b* locus. This result, obtained in a statistically independent population of animals, confirms that a QTL near the *b* locus accounts for about 40 percent of the genetic contribution to acute alcohol withdrawal (Crabbe et al. 1994*a*).

### LS x SS RI Strains: QTL Analyses in Animals Bred for Alcohol-Related Characteristics

The principles of QTL analysis also can be applied to the offspring of other genetic crosses, such as those derived from lines that have been bred selectively for their sensitivity to specific alcohol effects. Such analyses may enable researchers to identify more easily the QTLs related to the particular sensitivity. For example, RI’s were developed from the LS and SS mice, which were bred to differ with respect to alcohol-induced loss of righting reflex, that is, hypnotic sensitivity to alcohol.^6^ Erwin and Jones (1993) have examined 26 LS x SS RI strains for potential genetic influences on their hypnotic, hypothermic, and locomotor responses to alcohol. The researchers’ QTL analysis results suggest influences of several genes on these behaviors, with some of the genes appearing to influence more than one behavior.

In a similar project, Johnson and colleagues at the University of Colorado have begun to identify QTLs underlying the differential sensitivity to alcohol in the LS x SS RI’s. They recently found several potential QTLs that influence initial sensitivity to alcohol (personal communication; Markel et al. in press) and that currently are being evaluated in inbred LS and SS F_2_ mice.

## Summary

The use of QTL analysis has greatly extended current understanding of the particular genes involved in the expression of alcohol-related traits. Through the use of these statistical and molecular biological tools, made practical by an expanding library of known genetic markers, the complex genetic substrates of alcoholism can be examined even in the absence of prior knowledge or specific hypotheses. The suggested QTLs can then be evaluated further for their possible role in the expression of these traits.

## Footnotes

1 In this article, the term “alcoholism” refers to the criteria for alcohol abuse and dependence as outlined in the American Psychiatric Association’s *Diagnostic and Statistical Manual of Mental Disorders, Fourth Edition*.

2 For a definition of this and other technical terms used in this article, see central glossary, pp. 182–183.

3 Chromosomes are composed of strings of nucleotides; a gene is a specific sequence of nucleotides that influences a phenotype.

4 Correlation coefficients provide a measure of the likelihood that animals with a specific phenotype carry a particular marker in their genome.

5 Alcohol acceptance was measured by the amount of 10-percent alcohol in water a mouse consumed in the 24-hour period following 24 hours of fluid deprivation.

6 Following an injection of alcohol, mice lose their righting reflex (i.e., the reflex to point their bellies toward the ground and their backs up). This reflex is regained at different time periods after injection and is used as a measure of hypnotic sensitivity.
